# Editorial: Immunometabolic crosstalk during viral infection

**DOI:** 10.3389/fmicb.2024.1365507

**Published:** 2024-02-02

**Authors:** Wang Guo, Hengmi Cui, Xuming Hu

**Affiliations:** ^1^Jiangsu Key Laboratory for Animal Genetic, Breeding and Molecular Design, College of Animal Science and Technology, Yangzhou University, Yangzhou, Jiangsu, China; ^2^College of Animal Science and Technology, Institute of Epigenetics and Epigenomics, Yangzhou University, Yangzhou, Jiangsu, China; ^3^Joint International Research Laboratory of Agricultural and Agri-Product Safety, Ministry of Education of China, Yangzhou University, Yangzhou, Jiangsu, China

**Keywords:** immunometabolism, virus infection, immune response, glucose metabolism, glutamine metabolism

Immunometabolism has recently emerged as a key mechanism in regulating cell function and virus infection. For their survival and proliferation, viruses have evolved to employ the host's metabolic system (Li F. et al.; Wang et al.; Wu et al.). For example, using ultrahigh-performance liquid chromatography–quadrupole time-of-flight tandem mass spectrometry (UHPLC-QTOF-MS), Wang et al. found that 261 metabolites were significantly altered upon Marek's disease virus (MDV) infection (Wang et al.). Most changes occurred in amino acid, energy, nucleotide, and lipid metabolism (Wang et al.).

Viruses rely entirely on the host metabolic machinery to complete their life cycle and have evolved to rewire host cell glucose and glutamine metabolism. Wu et al. reported that C-C motif chemokine ligand 4 (CCL4) inhibits glucose metabolism and avian leukosis virus subgroup J (ALV-J) replication in chicken macrophages. ALV-J replication is dependent on the glucose metabolism, and inhibition of glucose metabolism by CCL4 overexpression could further limit ALV-J proliferation in macrophages. CCL4 can inhibit key gene expression of glucose metabolism, including MYC, hexokinase 1 (HK1), HK2, pyruvate kinase M2 (PKM2), and LDHA. Interestingly, CCL4 is negatively regulated by glucose metabolism and ALV-J infection. High glucose treatment or ALV-J infection significantly downregulated the expression of CCL4 in chicken macrophages. Therefore, regulation of immunometabolic crosstalk between the innate immune system and glucose metabolism during ALV-J infection may be an effective strategy to limit ALV-J infection.

Glutamine metabolism is essential for Siniperca chuatsi rhabdovirus (SCRV) replication, while aspartate metabolism plays an important role in viral proliferation in glutamine deficiency. Li F. et al. reported that the aspartate metabolic pathway was required for the replication and proliferation of SCRV in Chinese perch brain cells. SCRV infection enhanced the expression of key enzymes (including ASNS, MDH1/2, and GOT1/2) in the aspartate metabolic pathway. RNA interference-medicated knockdown of the expression of these key enzymes significantly impaired SCRV replication. Moreover, replication of SCRV is highly dependent on asparagine levels. When asparagine was added to the depleted medium, the SCRV copy number was restored to 90% of those in the replete medium. Wang et al. also found that MDV infection influenced the expression of the key enzyme GOT1 in the aspartate metabolic pathway. These findings suggest that the key enzymes of aspartate metabolic pathway may be a new target to effectively control viral infection.

Viruses have also developed multiple ways to interfere with the host's immune system (Li Z. et al.; Shi et al.; Xu et al.). Herpes simplex virus 1 (HSV-1) virus markedly inhibited interferon production by abrogating the nuclear translocation of phosphorylated NF-kappaB sub-unit p65 to escape the host antiviral response (Li Z. et al.). Goose astrovirus infection can activate pattern recognition receptors (RIG-I, MDA-5, TLR-3), thereby hindering virus invasion (Xu et al.). Shi et al. reviewed the molecular mechanisms of inflammasome (including NLRP1, NLRP3, and AIM2 inflammasomes) activation or inhibition by viruses, explored the crosstalk between inflammasome and other immune pathways, and developed novel inflammasome-based antiviral strategies. In addition, the gut virome could be an important part of the host's immune system and a novel antiviral target (Li J. et al.).

Immunometabolic crosstalk during viral infection will shed new light on how viruses regulate the intricate immune system via metabolic mechanisms. Future research should look into the crosstalk between immunometabolism and viral infection. Systemic immunometabolism induced by a viral infection will reveal new insights into how immune cells communicate with the organism to execute their diverse antiviral tasks most effectively. Ultimately, a central goal of the field is to apply immunometabolism findings to the discovery of novel antiviral strategies.

## Author contributions

WG: Writing—original draft, Writing—review & editing. HC: Writing—original draft, Writing—review & editing. XH: Writing—original draft, Writing—review & editing.

